# Strength and Durability Performance of Alkali-Activated Rice Husk Ash Geopolymer Mortar

**DOI:** 10.1155/2014/209584

**Published:** 2014-11-23

**Authors:** Yun Yong Kim, Byung-Jae Lee, Velu Saraswathy, Seung-Jun Kwon

**Affiliations:** ^1^Department of Civil Engineering, Chungnam National University, Daejeon 305-764, Republic of Korea; ^2^Corrosion and Materials Protection Division, CSIR-Central Electrochemical Research Institute, Karaikudi, India; ^3^Department of Civil Engineering, Hannam University, Daejeon 306-791, Republic of Korea

## Abstract

This paper describes the experimental investigation carried out to develop the geopolymer concrete based on alkali-activated rice husk ash (RHA) by sodium hydroxide with sodium silicate. Effect on method of curing and concentration of NaOH on compressive strength as well as the optimum mix proportion of geopolymer mortar was investigated. It is possible to achieve compressive strengths of 31 N/mm^2^ and 45 N/mm^2^, respectively for the 10 M alkali-activated geopolymer mortar after 7 and 28 days of casting when cured for 24 hours at 60°C. Results indicated that the increase in curing period and concentration of alkali activator increased the compressive strength. Durability studies were carried out in acid and sulfate media such as H_2_SO_4_, HCl, Na_2_SO_4_, and MgSO_4_ environments and found that geopolymer concrete showed very less weight loss when compared to steam-cured mortar specimens. In addition, fluorescent optical microscopy and X-ray diffraction (XRD) studies have shown the formation of new peaks and enhanced the polymerization reaction which is responsible for strength development and hence RHA has great potential as a substitute for ordinary Portland cement concrete.

## 1. Introduction

The production of one ton of cement emits approximately one ton of carbon dioxide to the atmosphere which leads to global warming conditions [1, 2]. It is important to reduce the CO_2_ emission through usage of waste materials like fly ash (FA), silica fume (SF), ground granulated blast furnace slag (GGBFS), and rice hush ask (RHA), which contributes to the improvement of the binder properties such as long-term strength, permeability, and durability [3–5]. The continuous increase in the generation of waste materials outlines a challenge to the investigators to propose solutions to its reuse [6, 7]. What is more, it is a habitual practice to incorporate these materials to hydraulic binders as a solution to their final confinement; besides that, in many occasions, the incorporation results in improvements of the properties of the resulting material as much in mechanical properties and durability [8, 9] Among the supplementary cementitious materials FA and GGBFS are widely used for high volume replacement for their enhancement of engineering properties [10, 11]; however, the other materials can be considered for reuse or reutilization of resources.

Rice husk constitutes 20% of the 500 million tons of paddy produced in the world. 75% organic volatile matter and the balance 25% of the weight of this husk are converted into ash during the burning process known as rice husk ash. RHA contains very high amount of silica content and is found to be amorphous in nature. The amorphous silica contained in RHA can react with cementitious binders to perform pozzolanic activity [12]. Geopolymer is an aluminosilicate material that exhibits very good strength, hardness, and chemical stability [13]. The chemical composition of geopolymer is similar to that of zeolite, but it shows an amorphous microstructure. Geopolymer binders have been reported as being acid-resistant [14], economical, environmentally-friendly, and more absorbent of liquids and produce a highly durable product [15]. Kusbiantoro et al. studied the effect of FA based geopolymer concrete and found that elevated temperature is suitable for rapid dissolution of silicate monomer and oligomer from microwave incinerated RHA surfaces, which supports the formation of supersaturated aluminosilicate solution in geopolymer system [16].

So far a systematic investigation of strength and microstructural and durability properties of RHA geopolymer concrete is very scarce. Hence, the present investigation aims to study the effect of RHA in making geopolymer concrete with different concentrations of sodium hydroxide with reference to strength characteristics, temperature effect, and microstructural and durability properties.

## 2. Experimental

### 2.1. Materials and Method

Ordinary Portland cement (OPC) conforming to ASTM C150-Type I was used for this study. River sand passing through 2.36 mm sieve having fineness modulus of 2.60 conforming to grading zone III was used for this investigation. The specific gravity and water absorption of fine aggregates were 2.41 and 0.5%, respectively. Sodium silicate was used as an alkali activator with sodium hydroxide solutions varying from 7 to 10 molar concentrations as shown in Table 1 which were used for the investigation.

**Figure 1:**
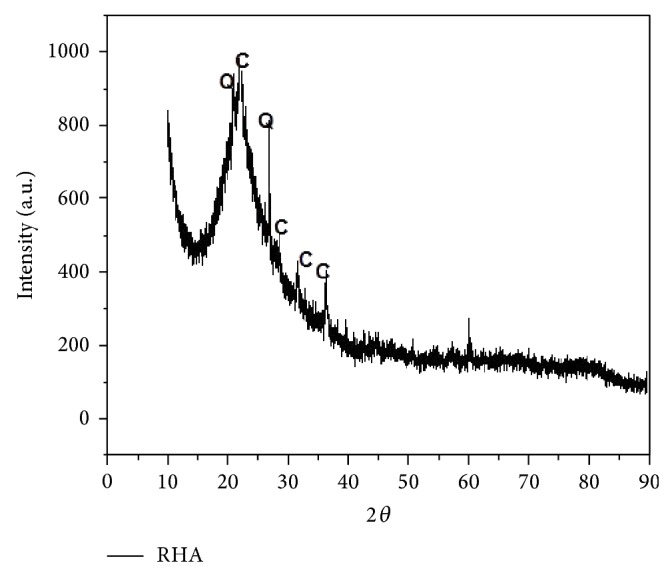
XRD pattern of RHA (C = cristobalite; Q = quartz).

RHA was obtained by burning rice husk at controlled temperature (650°C to 700°C) to achieve amorphous silica. XRD pattern of RHA used is given in Figure 1. Figure 1 shows that the diffraction peaks at 20.75° and 26.8° correspond to quartz and that other peaks at 21.7°, 28.9°, 31.2°, and 36° correspond to cristobalite. RHA was found to be in amorphous form between 2*θ* angle of 14.8° and 27.1°. The amorphous or crystalline formation of silica depends upon the temperature of burning and the method of ash production [17]. The chemical composition and physical properties of OPC and RHA are presented in Table 2.

### 2.2. Mix Design and Curing

**Figure 2:**
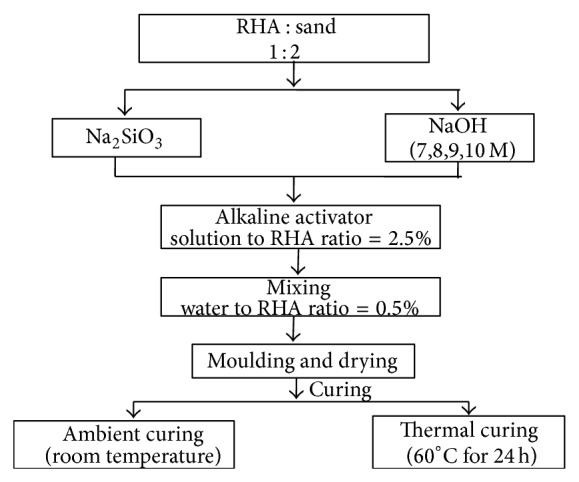
The mixing, casting, and curing procedures adopted for making geopolymer.

Geopolymer mortars were made with RHA : sand ratio 1 : 2. Sodium hydroxide concentrations used were 7, 8, 9, and 10 molar, and the sodium silicate to sodium hydroxide ratio 2.5% by mass was used. The mixing, casting, and curing procedures adopted for making geopolymer mortar are given in Figure 2. In thermal curing, the specimens were kept in oven at 60°C for 24 hrs and then were kept at room temperature over a period of 7, 14, and 28 days. For comparison, OPC mortar was cast with 0.45 W/C ratio and subjected to steam curing at 85% RH over a period of 7, 14, and 28 days. Then both the specimens were subjected to various types of investigations such us compression test, fluorescence microscopy, elevated temperature study, acid and sulfate resistance test, and ultrasonic pulse velocity test.

### 2.3. Compression Test

The compressive strength is one of the most important properties of mortar. Specimens of size of 100 × 100 × 100 mm cubes were cast with geopolymer mortar with different concentrations of NaOH. The specimens were subjected to ambient and thermal curing. For comparison, OPC mortar cubes were also cast and subjected to steam curing at 85% RH for 7, 14, and 28 days. At the end of curing, compression tests were conducted. For each system, triplicate specimens were cast. The cubes were tested in the compression testing machine of 60 T capacity. The load was applied at a rate of 140 kN/min. The ultimate load at failure was noted.

### 2.4. Fluorescence Microscopy and Capillary Flow Analysis

Fluorescence microscopy is an important tool to analyze concrete and mortar and other related building materials like stones, bricks, and so forth. Semitransparent thin sections as thin as 20 *μ*m of concrete can be analyzed by fluorescence microscopy in bright field or polarized light mode with or without tint plate. Additionally, the thin sections can be impregnated with the fluorescent epoxy for fluorescence microscopy. Examination with fluorescence light microscopy gives information about the porosity of the material. A dense area of the material appears in dark colour, while porous areas appear in light colour. Bright field microscopy reveals general information about the composition and structure of the material. Polarized light helps to identify the crystallinity and composition of the minerals. Microscopical examination of concrete helps to assure quality and to evaluate the durability and deterioration processes.

### 2.5. Capillary Flow Analysis

Cement mortar and geopolymer mortar specimens were cut into 1 Cm × 1 Cm × 1 Cm pieces and were treated with ethanol/acetone and kept in oven at 100°C for 24 hours to remove the free moisture present in the pores. Then the specimens were subjected to capillary flow analysis using capillary flow porometer (PMI Inc., USA) under a pressure of 250 psi to identify the pore size distribution and pore diameter of the samples.

### 2.6. Elevated Temperature Study

Muffle furnace was used for this study. Specimens were subjected to heat at temperatures of 300°C, 500°C, and 700°C at an incremental rate of 4°C per minute from room temperature. The temperature was sustained for 1 hour. Then the specimens were allowed to cool down for 24  hours at room temperature inside the furnace and tested for their compressive strengths after 7, 14, and 28 days.

### 2.7. Acid and Sulfate Resistance Test

Geopolymer mortars were subjected to acid and sulfate resistance test by immersing the specimens in 5% HCl, 5% H_2_SO_4_, 5% Na_2_SO_4_, and 5% MgSO_4_ over a period of 28 days and the weight loss was calculated. 5% is chosen on the basis of the aggressiveness when compared to 10% concentration.

### 2.8. Ultrasonic Pulse Velocity (UPV) Test

UPV measurements were carried out for all the geopolymer mortar cubes and the result was compared to control specimens. The velocity of an ultrasonic pulse is influenced by the properties of concrete which determines the elastic stiffness and mechanical strength. Hence, each material has typical ultrasonic pulse velocities. These velocities can be correlated with the material properties. Comparatively, higher velocity is obtained when concrete quality is good in terms of density, uniformity, and homogeneity. UPV measurements were also used to measure the defects and to assess the deterioration and soundness of the concrete [18].

## 3. Results and Discussion

### 3.1. Compressive Strength Test

**Figure 3:**
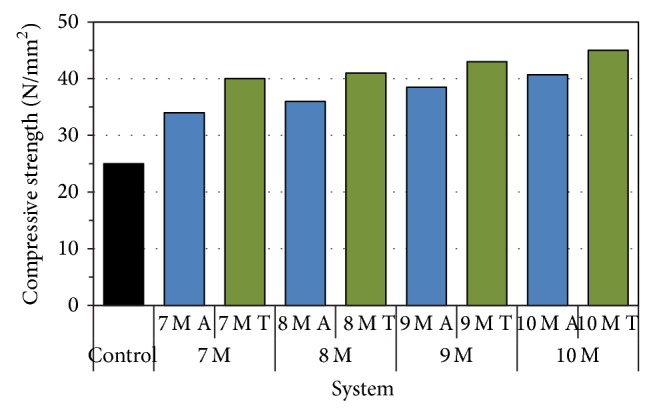
Compressive strength of geopolymer and control mortar at 28 days.

7-, 14-, and 28-day compressive strength that is obtained for geopolymer and control mortar cubes was reported in Table 3. From Table 3 it is observed that the compressive strength was found to increase with the increase in the curing period.  Figure 3 depicts the compressive strength of geopolymer and control mortar after 28 days of curing. From the figure it is observed that geopolymer mortar has shown higher compressive strength than control specimens. Thermally cured specimens have shown higher compressive strengths of 15% (7 M), 12.2% (8 M), 10.47% (9 M) and 9.6% (10 M), and 37.5% (7 M), 39% (8 M), 41.9% (9 M), 44.4% (10 M) after 28 days than the ambient cured and control samples. Among all, 10 M alkali-activated geopolymer concrete with thermal curing has shown higher compressive strength due to the presence of heat which accelerated the strength due to the strong Si-O-Al bond formation.

### 3.2. Fluorescence Microscopy

**Figure 4:**
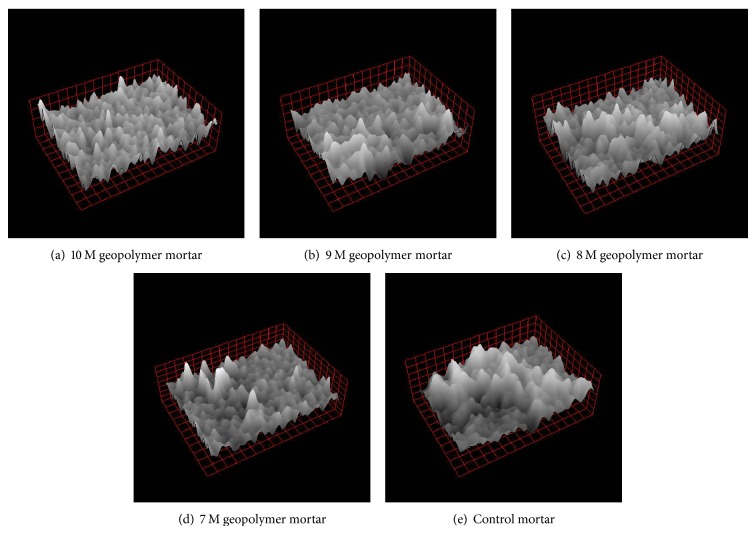
3D optical microscopic images of geopolymer and control mortars.

3D images of fluorescence micrographs taken for different molar concentrations of geopolymer mortars were given in Figures 4(a)–4(e). From the figures, it was found that as the concentration increases the height of the peak formation was found to be narrow and closer indicating the denser packing and as the concentration decreases the peaks were found to be short and broader indicating the porous nature of the mortar. At higher concentration, the particle packing was found to be dense and compact which is responsible for pore size refinement indicating the homogeneity of the geopolymer mortar.

### 3.3. Capillary Flow Analysis

**Figure 5:**
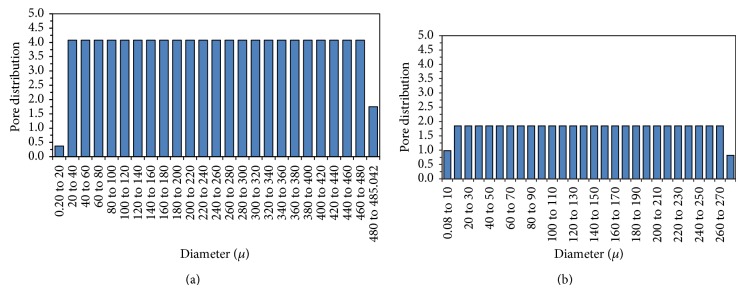
(a) Pore size distribution versus diameter of cement mortar. (b) Pore size distribution versus diameter of geopolymer mortar.

Figures 5(a) and 5(b) show the pore size distribution versus diameter of control and geopolymer (10 M) mortar. From the figure, it is observed that the geopolymer particles have lesser and uniform pore size distribution when compared to control mortar. The average pore diameter was ranging from 0.2 to 485 and from 0.08 to 274 *μ*m for control and geopolymer systems, respectively. From the data, it was observed that nearly 55% reduction in pore size distribution was observed for geopolymer mortar than the control mortar. This observation clearly confirmed the pore blocking effect due to the increasing in concentration of 10 M NaOH.

### 3.4. Elevated Temperature Study

**Figure 6:**
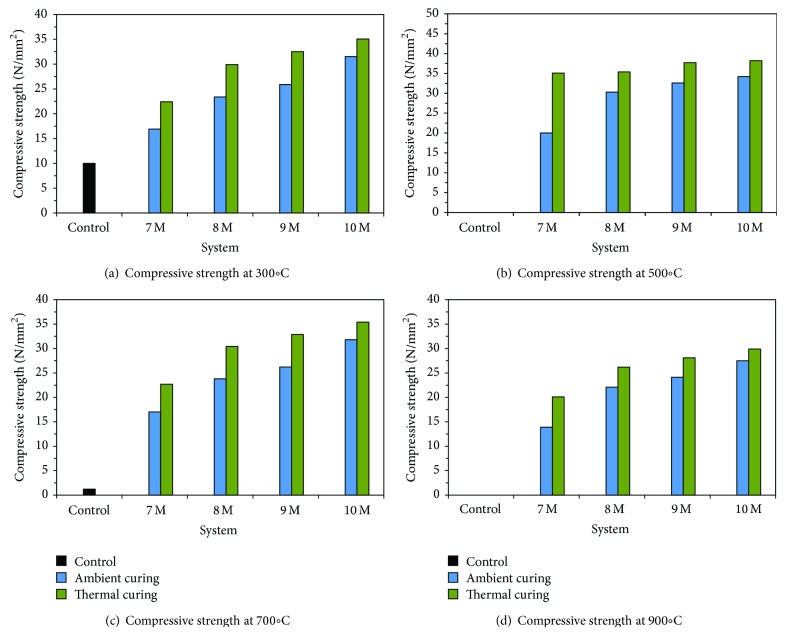
Compressive strength of various systems at elevated temperature.

Elevated temperature studies were carried out at 300°C and 500°C and at 700°C and 900°C for all the systems and represented in Figures 6(a)–6(d). From the studies, it is found that the compressive strength was found to decrease with the increase in temperatures due to the accelerated drying. Initially at low temperature (300°C) the strength loss of geopolymer mortars was observed to be very less due to the evaporation of surface moisture content. But the control specimens have lost 88% of their original compressive strengths at 300°C, and, beyond 300°C, the specimens got crumbled into powder indicating the total failure of the samples. As the temperature increases from 300°C to 900°C, evaporation of free water content was rapid and when it reaches the maximum limit, it creates pressure inside the geopolymer mortar, which leads to internal cracking due to vapour effect [19] that ultimately decreased the compressive of the specimens [20]. The presence of silicates caused swelling effect due to the thermal expansion which reduced the compressive strength at high temperature exposure [21].

### 3.5. Acid and Sulfate Resistance Test

**Figure 7:**
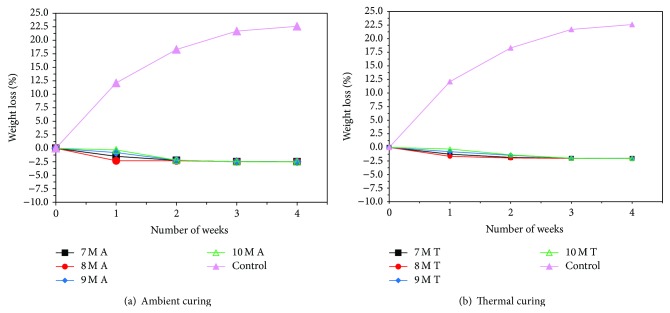
Weight loss for specimens immersed in 5% H_2_SO_4_ after 28 days.

**Figure 8:**
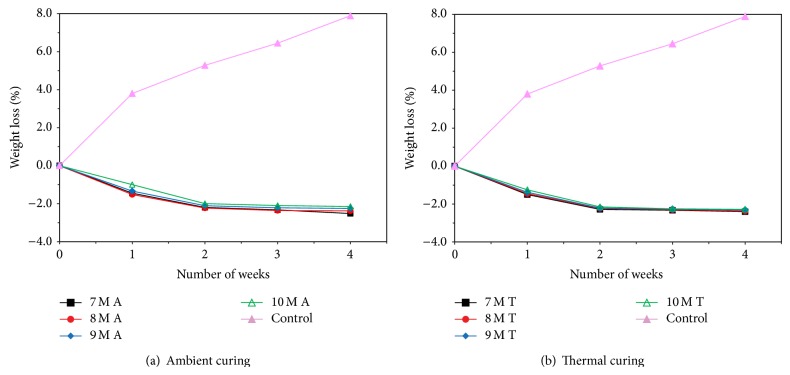
Weight loss for specimens immersed in 5% HCl after 28 days.

**Figure 9:**
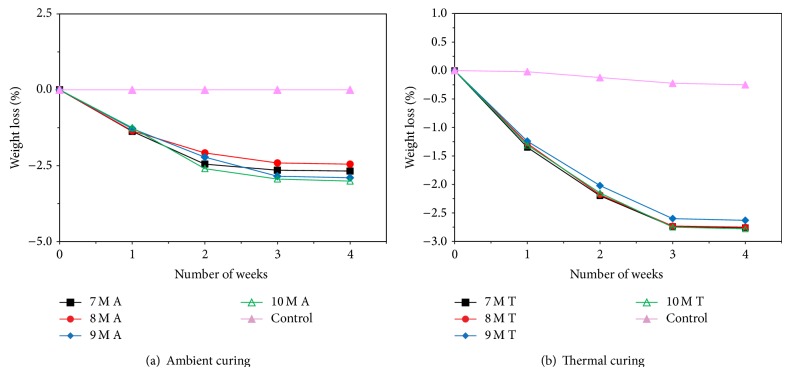
Weight loss for specimens immersed in 5% MgSO_4_ after 28 days.

**Figure 10:**
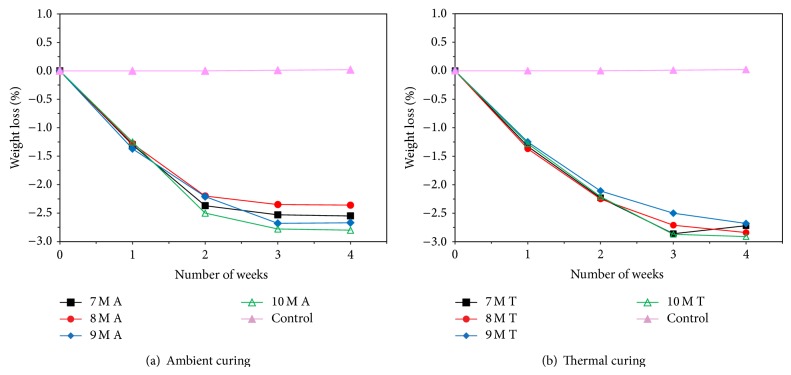
Weight loss for specimens immersed in 5% Na_2_SO_4_ after 28 days.

**Figure 11:**
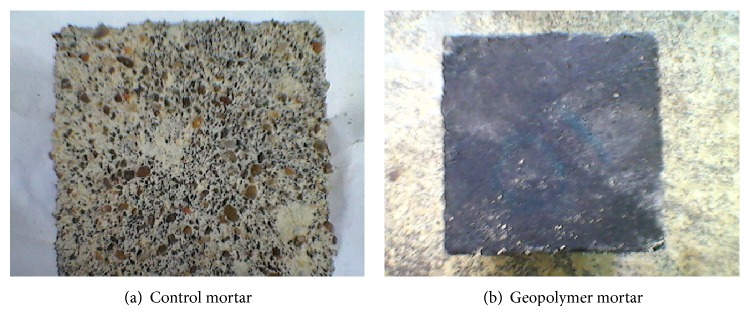
Photograph of specimen immersed in 5% H_2_SO_4_.

**Figure 12:**
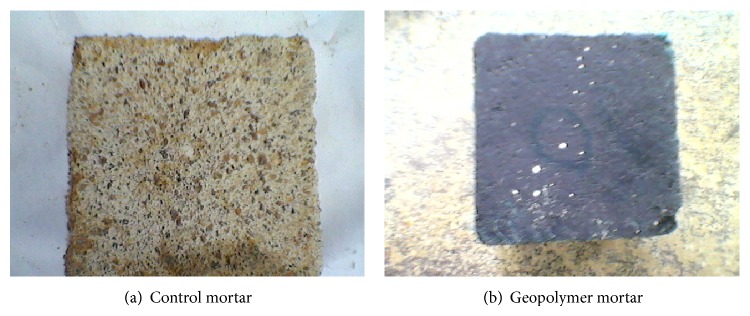
Photograph of specimen immersed in 5% HCl.

**Figure 13:**
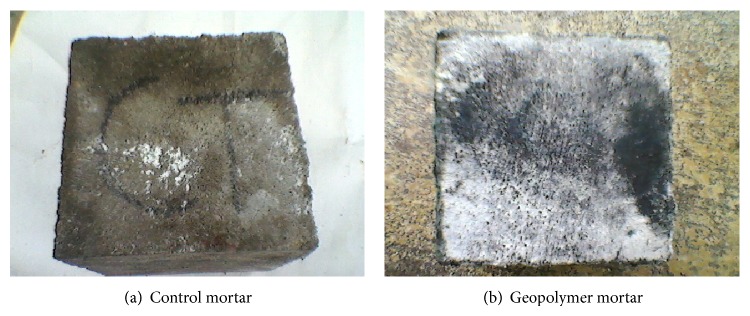
Photograph of specimen immersed in 5% N_2_SO_4_.

**Figure 14:**
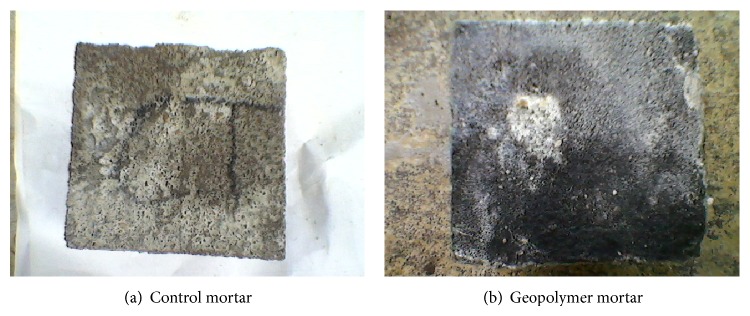
Photograph of specimen immersed in 5% MgSO_4_.

Figures 7, 8, 9, 10, 11, 12, 13, and 14 indicate the acid and sulfate resistance studies carried out for different geopolymer mortar and control specimens. From the studies, it is found that the percentage weight loss was found to be more than 22% and 8% in H_2_SO_4_ and HCl media, respectively. This weight loss is due to the presence of active calcium hydroxide. When compared to acid and sulfate immersion, acid is found to be aggressive, indicating higher weight loss. In general geopolymer mortar has excellent acid and sulfate resistance due to the absence of cement (Ca(OH)_2_). In acid and sulfate exposure, geopolymer mortars have shown weight gain of up to 2.5% after 28 days of exposure, which is due to scale formation in acid and deposition of white powder formation in sulphate exposure on the surface of the specimens [22, 23].

### 3.6. Ultrasonic Pulse Velocity Test

**Figure 15:**
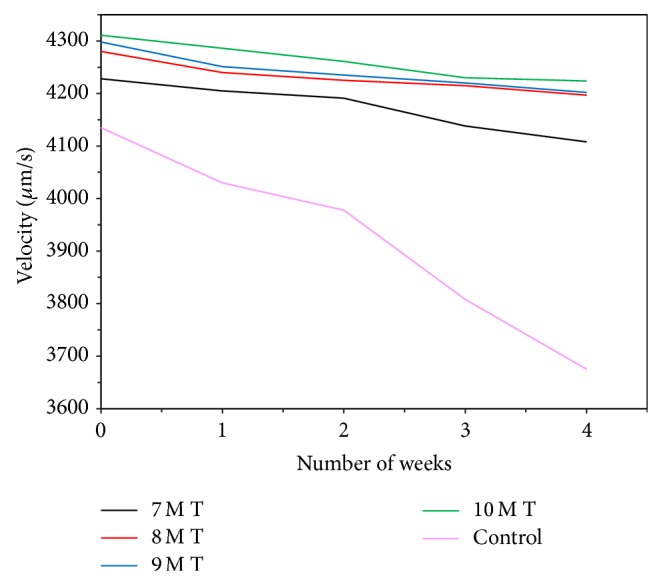
UPV for sulfuric acid.

UPV measurements were carried out for higher weight loss observed specimens.  Figure 15 shows the UPV measurements taken for thermal cured specimens immersed in 5% H_2_SO_4_ medium over a period of 4 weeks of exposure. Higher velocity reduction was observed for control specimens in H_2_SO_4_ solution than sulfate exposure. Control specimens have shown UPV values from 4138 to 3675 *μ*m/sec, whereas all the geopolymer systems have shown UPV values greater than 4100 *μ*m/sec, respectively, indicating the good quality of the mortar specimens even after 4 weeks of exposure in acid medium. It is well evidenced from the photographs of Figures 11(a) and 11(b) that the surface of the cement mortar specimens has shown etching of the surface by the dissolution of Ca(OH)_2_ particles present in the cement. Over long cycles of exposure, ettringite is formed. [24]. Ettringite is no longer stable and decomposes into aluminium hydrates and gypsum and calcium silicate hydrate becomes unstable. The formation of gypsum (calcium sulphate) leads to softening of the mortar, but geopolymer mortars are highly stable and acid-resistant because of the inert materials present in the mortar.

## 4. Conclusions

From the above investigation, the following broad conclusions were drawn.

1. Compressive strength test results indicated that alkali-activated RHA geopolymer mortars have shown superior performance to the control system.
2. Fluorescent microscopy studies revealed that geopolymer mortars have denser, compact, and homogenous particle packing of the mortar.
3. Capillary flow analysis indicated that geopolymer mortar has 55% reduction in pore diameter indicating the pore size refinement and pore blocking effect of the particles.
4. Elevated temperature studies indicated that all the geopolymer mortars are more resistant to temperature up to 900 degrees.
5. Acid immersion studies indicated that geopolymer mortars have shown better acid-resistant properties.
6. By considering the various points obtained from this investigation, RHA can be used as a substitute material for OPC.

## Figures and Tables

**Table 1:** Composition of alkali activators used for the investigation.

Key	Mass ratio of solution (1 : 2.5)			Composition of GP liquor			
	Na-silicate	NaOH	Total	Na_2_O	SiO_2_	H_2_O	Total
7 M	3.10	1.24	4.34	0.68	1.34	2.32	4.34
8 M	3.18	1.27	4.45	0.72	1.38	2.35	4.45
9 M	3.23	1.29	4.52	0.76	1.40	2.36	4.52
10 M	3.30	1.32	4.62	0.81	1.43	2.38	4.62

**Table 2:** Chemical composition and physical properties of OPC and RHA.

Compound (%)	OPC	RHA
SiO_2_	20.90	90.79
Al_2_O_3_	5.40	2.22
Fe_2_O_3_	4.60	0.80
CaO	63.50	0.92
MgO	0.60	0.47
SO_3_	2.60	—
Na_2_O	0.15	0.50
K_2_O	0.25	0.30
LOI	2.00	4.00
Specific gravity	3.15	2.26
Average particle size (*μ*)	45.34	25.42
Colour	Grey	Grey

**Table 3:** 7-, 14-, and 28-day compressive strength of geopolymer and control mortar.

Number of days	Compressive strength of geopolymer mortar (MPa)								
	Concentration of alkali activator/curing								
	7 M		8 M		9 M		10 M		Control
	A	T	A	T	A	T	A	T	
7	9	19	13	21	16	22	17	26	10
14	19	27	19	28	22	30	23	31	19
28	34	40	36	41	38.5	43	40.7	45	25

A: ambient curing; T: Thermal curing.
